# Editorial

**Published:** 1854-12

**Authors:** 


### THE MEDICAL EXAMINER.

### PHILADELPHIA, DECEMBER, 1854.

We have been obliged to defer the notice of several works with which we have been favored, as the original articles in the present number occupy an unusual amount of space. Dr. H. H. Smith's case of *Ovariotomy* will be published in the January number.

*Abstract of Meteorological Observations for October*, 1854, *made at Philadelphia, Pa. Latitude* 39° 57′ 28″ *N., Longitude* 75° 10′ 40″ *W. from Greenwich.* By PROF. JAMES A. KIRKPATRICK.

| 1854. Oct. | Barometer. Daily Mean | Barometer. Mean Daily Range. | Thermom. Daily Mean | Thermom. Mean Daily Range | Dew Point 2 P. M. | Rel. Humid. 2 P. M. | Rain. | Prevailing Winds. | General Remarks. |
|---|---|---|---|---|---|---|---|---|---|
| | Inches. | Inches. | Deg. | Deg. | Deg. | Hunds. | Inch. | Points. | |
| 1 | 29.936 | .309 | 60.0 | 5.7 | 48.0 | 0.56 | 0.015 | SW. | Cloudy; ev. rain. |
| 2 | 29.779 | .207 | 65.0 | 5.7 | 40.0 | .42 | | (Var.) | Cloudy. [*lowest* 29.439. |
| 3 | 29.557 | .221 | 68.0 | 3.0 | 67.7 | .94 | 0.268 | S. | Cloudy; rain 11 A.M to 3 P.M. *Bar.* |
| 4 | 29.694 | .228 | 62.3 | 7.0 | 42.7 | .50 | 0.246 | (Var.) | M. rain; aft. and ev. clear. |
| 5 | 30.137 | .443 | 55.2 | 7.2 | 35.7 | .43 | | W.NW. | Cloudy. |
| 6 | 30.300 | .163 | 56.0 | 6.2 | 45.0 | .54 | | (Var.) | M. fog; aft. and ev. clear. |
| 7 | 30.108 | .158 | 63.5 | 7.5 | 52.0 | .59 | | SW. | Clear. |
| 8 | 30.019 | .094 | 68.7 | 5.2 | 50.0 | .50 | | SW. | Clear. |
| 9 | 29.931 | .088 | 71.3 | 3.3 | 44.0 | .39 | | SW. | Clear. *Therm. highest* 79°. |
| 10 | 29.984 | .102 | 67.3 | 4.0 | 46.7 | .52 | | (Var.) | M. cloudy; aft. and ev. clear. |
| 11 | 30.035 | .095 | 67.0 | 2.3 | 57.0 | .71 | | (Var.) | Cloudy. |
| 12 | 29.875 | .160 | 73.7 | 6.7 | 54.7 | .57 | 0.227 | SW. | Cloudy; rain at night. |
| 13 | 29.842 | .080 | 68.7 | 5.0 | 55.7 | .67 | | (Var.) | Cloudy. |
| 14 | 29.630 | .212 | 64.7 | 5.7 | 62.7 | .94 | 0.287 | E.SW. | M. and aft. rain; ev. clear. |
| 15 | 29.605 | .115 | 46.0 | 18.7 | 34.0 | .56 | | NW. | M. and ev. clear; aft. cloudy. |
| 16 | 29.611 | .008 | 49.0 | 3.7 | 40.0 | .62 | | W.NW. | M. and ev. clear; aft. cloudy. |
| 17 | 29.785 | .174 | 49.0 | 2.0 | 37.7 | .56 | | W. | Cloudy. |
| 18 | 29.975 | .190 | 47.7 | 4.0 | 33.7 | .41 | | W. | M. and ev. clear; aft. cloudy. |
| 19 | 30.232 | .257 | 43.0 | 5.3 | 29.3 | .43 | | NW. | Clear. [*lowest* 34°. |
| 20 | 30.320 | .088 | 45.2 | 4.2 | 38.3 | .64 | | (Var.) | Cloudy. *Bar. higest* 30.330; *Therm.* |
| 21 | 30.289 | .031 | 48.8 | 3.7 | 37.3 | .51 | | NE. | Clear. |
| 22 | 30.203 | .083 | 49.3 | 0.8 | 37.0 | .47 | | N.NW. | Clear. |
| 23 | 30.206 | .028 | 53.3 | 4.0 | 38.7 | .46 | | NE. | Clear. |
| 24 | 30.178 | .040 | 52.5 | 0.8 | 38.2 | .45 | | N. | Clear. |
| 25 | 30.225 | .057 | 57.8 | 5.3 | 45.7 | .52 | | N. | Clear. |
| 26 | 30.285 | .060 | 58.8 | 3.0 | 48.3 | .60 | | N. | Clear. |
| 27 | 30.232 | .053 | 58.7 | 2.2 | 48.0 | .56 | | N. | Clear. |
| 28 | 30.132 | .100 | 60.7 | 6.3 | 59.7 | .94 | 0.597 | NE. | Cloudy; rain all day. |
| 29 | 30.007 | .122 | 69.3 | 9.0 | 64.7 | .81 | 0.157 | SE. | Cloudy; aft. rain. |
| 30 | 29.924 | .086 | 67.2 | 2.2 | 65.0 | .92 | 0.061 | NE. | Cloudy; aft. and night rain. |
| 31 | 29.775 | .146 | 66.0 | 2.2 | 65.3 | .90 | 0.060 | SW. | M. and aft. rain; ev. clear. |
| Means for Oct. 1854 | 29.994 | .135 | 59.1 | 4.9 | 47.2 | .60 | 1.918 | N. 70° W. 32—100. | |
| Means for Oct. 1853 | 29.974 | | 53.2 | | 39.9 | | 3.470 | | |
| Means for Oct. 1852 | 29.976 | | 58.3 | | 48.0 | | 2.250 | | |
| Means for Oct. 3yrs | 29.981 | | 56.9 | | 45.0 | | 2.546 | N. 61½° W. 42—100. | |

The extreme range of the barometer during the month was 0.891 of an inch, and of the thermometer 45°.

